# Mapping Autism in Armenia Among Children and Adolescents Aged 0–18 Years: Population-Based Insights into Prevalence and Its Geographical Differences

**DOI:** 10.3390/children13010035

**Published:** 2025-12-26

**Authors:** Aram Hayrapetyan, Naira Khachikyan, Armine Aslanyan, Armen Mkrtchyan, Armenuhi Qotanyan, Meri Mkhitaryan, Ganna Sakanyan, Tamara Avetisyan, Sofya Mkrtchyan, Larisa Avetisyan, Konstantin Yenkoyan

**Affiliations:** 1 Department of Hygiene and Ecology, Yerevan State Medical University after Mkhitar Heratsi, 2 Koryun str, Yerevan 0025, Armenia; aramsurg@hotmail.com (A.H.); nairakhachikyan5@gmail.com (N.K.); aslanyanah@gmail.com (A.A.); armenmkrtchyan76@gmail.com (A.M.); armin-qot@mail.ru (A.Q.); sofa.mkrtchyan31@gmail.com (S.M.); larisaravetisyan@gmail.com (L.A.); 2 Cobrain Center, Yerevan State Medical University after M. Heratsi, 2 Koryun str, Yerevan 0025, Armenia; tamara.avetisyan.1970@gmail.com; 3 Neuroscience Laboratory, Cobrain Center, Yerevan State Medical University after M. Heratsi, 2 Koryun str, Yerevan 0025, Armenia; meri_mkhitaryan15@alumni.aua.am; 4 Department of Public Health and Healthcare Organization, Yerevan State Medical University after M. Heratsi, 2 Koryun str, Yerevan 0025, Armenia; sganna24@mail.ru; 5 Muratsan University Hospital Complex, Yerevan State Medical University after M. Heratsi, 114 Muratsan St., Yerevan 0075, Armenia

**Keywords:** autism spectrum disorder, Armenia, prevalence, urban, rural, male, female, ratio, mean age at diagnosis

## Abstract

**Highlights:**

**What are the main findings?**

- This study provides the first nationwide, population-based estimate and geographic mapping of autism spectrum disorder (ASD) prevalence among children and adolescents in the Republic of Armenia.
- ASD prevalence was higher among males, in urban areas, and in the capital city, Yerevan, with a mean age at diagnosis of 4.5 years.

**What are the implications of the main findings?**

- The observed geographic and urban–rural differences suggest variation in ASD detection and diagnosis, likely related to healthcare access and service availability.
- These findings establish a national baseline to support health policy development, service planning, and future epidemiological, etiological, and biologically informed research, potentially contributing to more targeted and personalized approaches to ASD care.

**Abstract:**

**Background/Objectives:** This study aimed to estimate the prevalence of autism spectrum disorder (ASD) among children and adolescents aged 0–18 years in the Republic of Armenia (RA), characterize geographic and sex-specific variations, and determine the mean age at diagnosis. These data are essential for informing health system planning, evaluating potential disparities in access to diagnostic services, and generating hypotheses regarding the biological and healthcare-related factors underlying geographic variation in ASD prevalence. **Methods:** A cross-sectional study was conducted using administrative records from outpatient medical facilities serving children and adolescents across all ten marzes of RA and the city of Yerevan in 2021. ASD prevalence was estimated at the national and regional levels and stratified by urban and rural residence. The male-to-female prevalence ratio and the mean age at ASD diagnosis were also calculated. **Results:** The overall prevalence of ASD in RA was 2.3 per 1000 children (95% CI: 2.1–2.4). The highest prevalence was observed in Yerevan (3.7 per 1000). The mean age at diagnosis was 4.5 ± 2.5 years. Males exhibited a 5.1-fold higher prevalence compared to females. ASD prevalence was significantly higher in urban areas than in rural settings. **Conclusions:** ASD prevalence in RA demonstrates marked geographic and sex disparities, with significantly higher rates in urban regions and among males. These findings underscore the importance of continued ASD prevalence monitoring in RA to identify trends, explore potential biological and healthcare-related contributors to geographic variation, and improve healthcare system planning and management. In turn, improved understanding of regional differences in ASD prevalence may help inform more targeted and personalized approaches to care. The results may also support national and regional policymakers in health, education, and related sectors in developing and strengthening services for children and adolescents with ASD and their families.

## 1. Introduction

Autism spectrum disorder (ASD) is a neurodevelopmental disorder that is characterized by persistent deficits in the ability to initiate and sustain reciprocal social interaction and social communication and by a range of restricted, repetitive, and inflexible patterns of behavior, interests, or activities that are clearly atypical or excessive for the individual’s age and sociocultural context [1].

The etiology and exact pathogenesis of ASD remain unclear [2]. Genetic and environmental factors, as well as their interactions, are widely discussed as risk factors of ASD [3,4,5]. Though scientists emphasize the role of genetic factors [6,7], there is a growing interest in the evaluation of environmental contribution to ASD [8,9,10,11], which includes pre-, post-, and neonatal factors, as well as environmental chemicals [12]. For instance, advanced parental age [13,14,15], self-reported stress during pregnancy, pregnancy with complications, short intervals between pregnancies [16], low birth weight [17,18], small gestational age [17], etc., are associated with significantly increased risk of ASD.

As it is mentioned above, ASD starts in early childhood and lasts throughout a person’s life, leading to psychological, emotional, and financial burden on family, caretakers, and the country. While there is no specific cure for ASD, starting therapeutic interventions as early as possible can lead to improved outcomes in social skills, daily living abilities, and overall quality of life for children with ASD [19].

The prevalence of ASD cases varies across the world, which is likely conditioned by the methodological differences in ASD case detection and case definition, as well as by the continuous increase in worldwide prevalence estimates. The main sources of ASD cases are administrative data, ad hoc studies, and surveys based on questionnaires. Studies have shown that when ASD prevalence estimates are based on ASD cases determined by teachers’ or parents’ reports, prevalence values are significantly overestimated as compared to the administrative data based on professional diagnoses [20]. Though the ASD prevalence estimates are characterized by high variability in different countries, there is also a constant tendency: ASD prevalence increased in the majority of countries over the last few decades. The World Health Organization (WHO) reported that about 1 in 160 children had autism in 2013 [21], while in 2021, this diagnosis was already revealed in about 1 child in 127 [22]. At the same time, ASD prevalence is higher among boys than girls globally [23]. Nevertheless, the male-to-female ratio estimates vary across the world.

In the United States, ASD prevalence estimates increased from 6.7 per 1000 children in 2000 to 27.6 per 1000 in 2020 [24]. In Sweden, ASD prevalence rose from 4.2 per 1000 in 2001 to 14.4 per 1000 in 2011 [25]. In Germany, the prevalence of ASD among individuals aged 0–24 years increased from 0.22% in 2006 to 0.38% in 2012 [26]. In Canada, according to national estimates from the Public Health Agency of Canada, 1.5% of children and youth aged 5–17 years had been diagnosed with ASD in 2018 [27], with prevalence rising to 2.0% among children and youth aged 1–17 years in a subsequent national report published in 2022 [28].

What is more, the trend of rising ASD estimates can be explained not only by an increase in the number of cases but also due to better diagnosis, greater awareness related to the mentioned pathology, etc.

To our knowledge, there has not been a large-scale study conducted on the prevalence of ASD in the Republic of Armenia (RA). Moreover, there is no unified national registry for ASD prevalence in the Republic of Armenia. In routine outpatient healthcare reporting, ASD cases are not recorded as a separate diagnostic entity but are instead included within the broader category of mental and behavioral disorders. This aggregation substantially complicates the systematic collection, extraction, and analysis of ASD-specific epidemiological data and largely explains the absence of previously published nationwide prevalence estimates. Consequently, dedicated population-based investigations are required to generate reliable and policy-relevant ASD prevalence data.

Thus, the aim of this study was to conduct the first nationwide, population-based assessment of autism spectrum disorder (ASD) prevalence among children and adolescents aged 0–18 years in the Republic of Armenia. Specifically, the objectives were as follows:

(i) To estimate the prevalence of ASD at the national level, including comparisons between urban and rural areas and across administrative regions;
(ii) To develop an initial geographic mapping of ASD prevalence throughout the Republic of Armenia;
(iii) To determine the male-to-female prevalence ratio;
(iv) To estimate the mean age at ASD diagnosis nationwide.

By addressing these objectives, this study establishes a foundational epidemiological framework to support future research and provides evidence to inform health policy, service planning, and targeted interventions for children and adolescents with ASD and their families.

## 2. Methods

This study represents the first child population-based prevalence investigation conducted across all ten marzes (“administrative provinces”) and Yerevan city in Armenia.

A cross-sectional study was undertaken, encompassing approximately 80% of the child population aged 0–18 years in the RA in 2021.

As there is no unified registry for ASD prevalence in the RA, the only source of consistent and reliable data for monitoring ASD prevalence is through the registries of outpatient clinics. This investigation utilized existing administrative databases from records of outpatient medical facilities catering to children and adolescents aged 0–18 years in all ten marzes and Yerevan in 2021. The study population comprised children and adolescents aged 0–18 years who were registered in outpatient medical facilities throughout RA from 1 January to 31 December 2021. The 10 marzes of the RA include Aragatsotn, Ararat, Armavir, Gegharkunik, Kotayk, Lori, Shirak, Syunik, Tavush, and Vayots Dzor marz.

**Figure 1:**
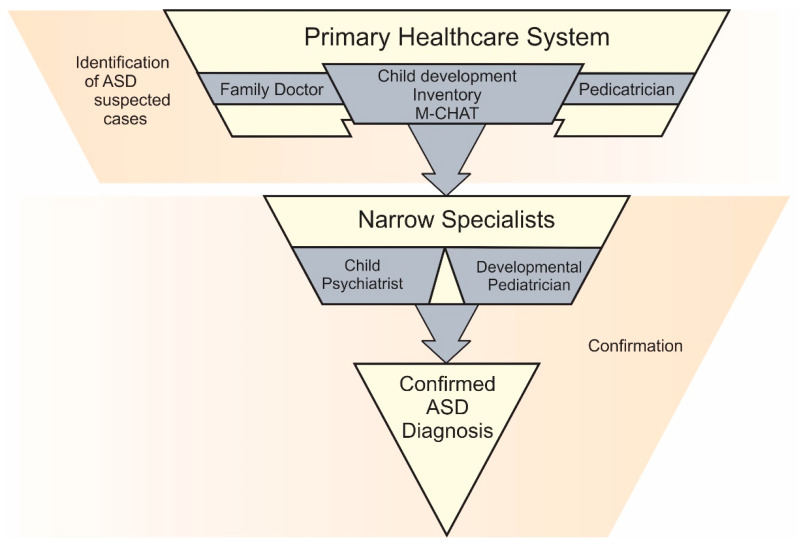
Stages of ASD diagnosis in RA.

In the outpatient clinic registries of RA, the former estimate focused on Autism Disorder (AD). The latter estimate encompassed a broader category defined as pervasive developmental disorder (PDD). This categorization follows the diagnostic criteria of [29] under the code F84. This code includes the following: F84.0: autistic disorder; F84.2: Rett’s syndrome; F84.3: other childhood disintegrative disorder; F84.5: Asperger syndrome; F84.8: other pervasive developmental disorders (PDD); F84.9: PDD, unspecified. All cases of AD and PDD were collectively classified as cases of ASD. As there are no specific biomarkers for ASD determination, the diagnosis is typically based on expert judgement. All individuals who were considered as suspected cases of ASD by a pediatrician/family doctor, based on the results of the Modified Checklist for Autism in Toddlers (M-CHAT) and the results of child development inventory by Harold Ireton (completed by parents and assessed by pediatrician/family doctor) [30], were evaluated by a child psychiatrist and a developmental pediatrician (Figure 1). A child or adolescent was defined as a confirmed case of ASD only after confirmation by the child psychiatrist and developmental pediatrician. What is more, all suspected cases of ASD were examined by the same group of specialists throughout RA.

The prevalence of ASD in 2021 was estimated comprehensively across the RA, including individual assessments for each of the 10 marzes and Yerevan city, as well as separate evaluations for urban and rural areas. The male-to-female ratio was calculated for the entire RA across 10 marzes and Yerevan city for both urban and rural settings. Additionally, the average age at diagnosis was estimated for all the mentioned categories.

Data input and analysis were conducted using the Statistical Package for Social Sciences (SPSS, version 23) and Microsoft Excel. The results obtained from processing the data were presented as measures of descriptive statistics: rates for qualitative variables and arithmetic means with standard deviations for quantitative ones. ASD prevalence was calculated as the number of children and adolescents with confirmed ASD divided by the total number of child population registered in the corresponding outpatient medical facilities in RA in 2021 per 1000, and a 95% confidence interval has been estimated. The normal approximation was used to estimate confidence intervals for prevalence, as the sample sizes were large, and there was no difference between the calculated and the exact Poisson confidence intervals.

Parametric tests were used to assess the statistical significance of differences between arithmetic means, given the normal distribution of variables: the t-test for independent samples when comparing two means and the Brown–Forsythe test for comparing more than two groups due to unequal variances among the compared groups. The null hypothesis was rejected at a significance level of *p* < 0.05. The statistically significant association was considered when *p* < 0.05.

## 3. Results

In 2021, a total of 1217 children and adolescents aged 0 to 18 years were diagnosed with ASD and recorded in outpatient medical facilities across all ten marzes and Yerevan city in the RA (Table 1). This study yielded an overall prevalence estimate of 2.3/1000 (95% CI 2.1–2.4). The mean age at the time of ASD diagnosis was calculated to be 4.5 ± 2.5 years old. The ages at diagnosis ranged from a minimum of 1 year to a maximum of 16 years.

The investigation into the sex ratio of ASD prevalence revealed a significantly higher prevalence rate of ASD among male children and adolescents compared to their female counterparts. Specifically, ASD was found to be 5.1 times more prevalent in males than in females. The prevalence rate for males was recorded at 3.6 per 1000 individuals (95% CI 3.4–3.8), whereas for females, it was 0.8/1000 (95% CI 0.7–0.9) (Table 2).

**Figure 2:**
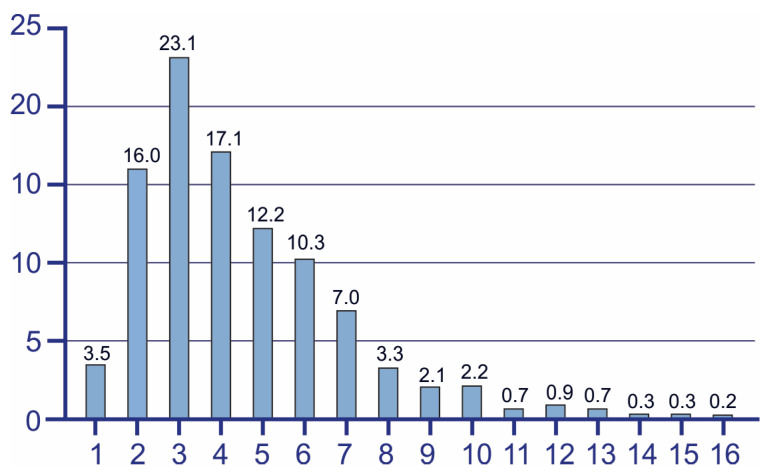
Age of ASD diagnosis in RA. Figure illustrates the distribution of ages at which ASD diagnosis was confirmed during the study period.

Analyses of the age at diagnosis show that the highest prevalence was observed in the age range from 2 to 6 years old (Figure 2). Among younger children, the number of confirmed ASD cases increases drastically, reaching the peak at the age of 3. From this point onwards, prevalence begins to decline gradually. Specifically, 19.5% of diagnoses were confirmed in children aged up to 2 years, 62.7% of ASD cases were identified before the start of school education, and the rest 17.8% of ASD cases were diagnosed in older age groups.

**Figure 3:**
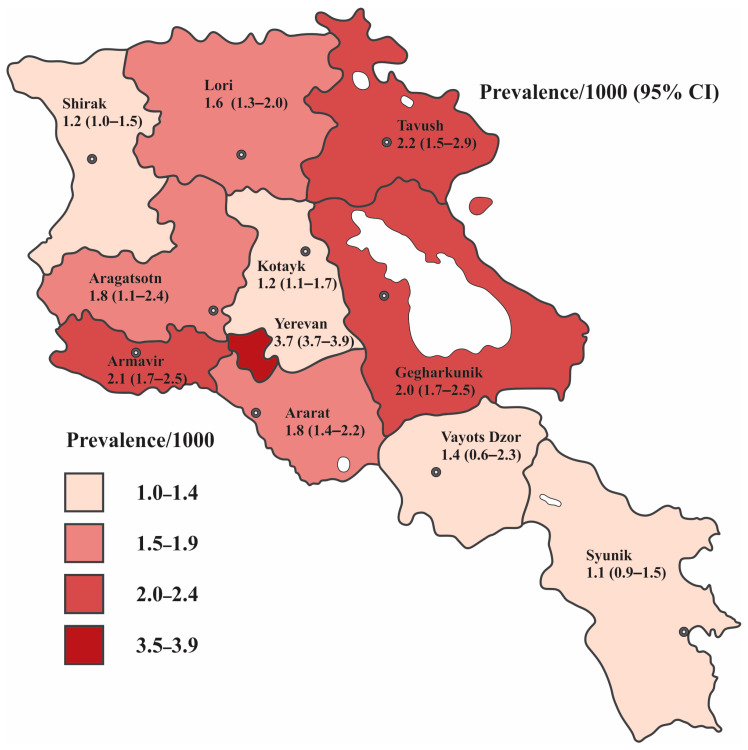
ASD prevalence estimates per 1000 children and adolescents aged 0–18 years registered in 10 regions and Yerevan, the capital city of RA, 2021. The dark dot with a white center shows the geographical location of the marz center.

The variation in prevalence estimates in the children population between marzes and Yerevan city was marked, ranging from 1.1/1000 to 3.7/1000, as represented in Table 3 and illustrated in Figure 3. The highest rate of ASD comes from Yerevan, estimating the prevalence of 3.7/1000. This was followed by Tavush, Armavir, and Gegharkunik marzes, which had closely comparable prevalence estimates of 2.2/1000, 2.1/1000, and 2.0/1000, respectively. The prevalence in Aragatsotn and Ararat was equal (1.8/1000). Lori and Vayots Dzor reported rates of 1.6/1000 and 1.4, respectively. The prevalence estimates in Kotayk, Shirak, and Syunik were relatively similar and recorded at 1.2/1000, 1.2/1000, and 1.1/1000, respectively.

The prevalence rate of ASD in Yerevan was significantly higher compared to the prevalence rates across all RA marzes. Table 4 provides the prevalence ratios along with their 95% confidence intervals.

Although ASD was consistently more prevalent among boys throughout Armenia, the male-to-female ratio varied significantly by region (Table 3). The highest male-to-female ratios were observed in Gegharkunik, Ararat, Syunik, and Kotayk, with ratios of 6.9:1, 6.8:1, 6.7:1, and 5.9:1, respectively. In Yerevan, the ratio was 5.2:1, closely followed by Lori (5.0:1) and Shirak (4.9:1). In Armavir, boys were 4.4 times more likely to be diagnosed with ASD compared to girls. In Tavush, this ratio was 3.7:1, and in Aragatsotn, it was 3.3:1. Vayots Dzor reported the lowest male/female ratio, with the prevalence among boys being nearly equal to that among girls (1.2:1).

Table 5 shows that the lowest average age at diagnosis is registered in Gegharkunik (3.41 ± 1.75 years old), while the highest one is in Lori (4.77 ± 2.34 years old). At the same time, the performed Brown–Forsythe test showed that the differences between the marzes were not significant (F = 1.779, *p* = 0.066).

The prevalence of ASD among children and adolescents residing in urban and rural areas has been documented as 3.0/1000 and 1.0/1000, respectively (Table 6). The difference between the indicated rates was statistically significant. The condition was found to be 5.4 times more prevalent among male children and adolescents than among females in urban areas and 3.8 times higher among boys in rural settings.

The mean age of confirmed ASD diagnosis was calculated as 4.27 ± 2.44 years in rural areas and 4.58 ± 2.56 years in urban areas (Table 7); however, the difference was not statistically significant (t = 1.551, *p* = 0.121).

## 4. Discussion

Our study is the first (to our knowledge) population-based investigation of ASD prevalence in children and adolescents aged 0–18 years across all 10 RA marzes and Yerevan city. A cross-sectional study was conducted, including approximately 80% of the child population aged 0–18 years from RA in 2021. ASD prevalence was calculated based on the data related to confirmed cases of ASD, registered in existing administrative databases of outpatient medical establishments related to children/adolescents aged 0–18 years in 2021 from all 10 marzes and Yerevan city of Armenia.

The results of different studies show high variability of ASD prevalence estimates worldwide. ASD prevalence estimate is reported as 27.6/1000 in the USA [24], 20/1000 in Canada [28], 17.6/1000 in England [31], 14.4/1000 in Sweden [25], 12.3/1000 in Spain [32], and 9.5/1000 in Italy [33]. In France, Finland, Denmark, and Iceland, ASD prevalence estimates were registered at 4.8/1000 to 7.3/1000, 7.7/1000, 12.6/1000, and 31.3/1000, respectively [34]. In some European countries, ASD prevalence estimates are registered at lower values. For instance, the prevalence of ASD in Germany was estimated at 3.8/1000 [26]; in Poland, it was 3.5/1000 [35], etc. While the ASD prevalence in Asia was reported as 3.6 per 1000 [36], estimates of ASD prevalence in Asian countries, like in Europe, also vary widely. The prevalence of ASD was recorded as 10.8 per 1000 in China [37], 7.52 per 1000 in Vietnam [38], and 6.55 per 1000 in Israel [39]. In India, estimates range from 1.5 per 1000 [40] to 2.3 per 1000 [41], etc.

The results of our study have shown an overall ASD prevalence estimate of 2.3/1000. The highest ASD prevalence is registered in the capital of RA—Yerevan, yielding 3.7/1000. This may be influenced by increased awareness campaigns and educational programs about ASD among healthcare professionals and the general population; greater acceptance of ASD; improved access to specialized healthcare services for ASD, resulting in more diagnoses; higher population density facilitating more interactions and diagnoses; and families with children diagnosed with ASD relocating from rural to urban areas for better services, among other factors. What is more, all child/adolescent psychiatrists and developmental pediatricians, confirming the ASD diagnosis, are located in the Yerevan medical healthcare system. Our findings in the Armenian children population show a prevalence of ASD that is twice as high as the estimate in the regional neighborhood, such as for 6–18 year olds in Iran, which is reported as 1.0 per 1000 [42], but lower compared to corresponding estimates in 8–10 year-old children in Georgia, which are reported as 4.25% [43].

In terms of sex distribution, our findings indicate that ASD is 5.1 times more prevalent among male children and adolescents compared to females. Our study’s findings mirror those of the study conducted in Israel, where the male-to-female ratio was reported as 5.1 to 1 [39]. In certain reports, such as among children in Taiwan from 2004 to 2010, the male-to-female ratio was higher, ranging from 5.64 to 6.06 to 1 [44]. Other studies have reported lower male-to-female ratios, such as 4.6 to 1 in Turkey [45], 4.5 to 1 in Spain [32], 4.32 to 1 in England [31], 4.25 to 1 in Egypt [46], 3.8 to 1 in the USA [24], and 2 to 1 in Iran [42]. In Finland, Denmark, southeast France, Iceland, and southwest France, sex ratios were reported as 3.3, 3.9, 4.0, 4.4, and 5.4, respectively [34]. On a global scale, the median male-to-female ratio was 4.2 to 1 according to a literature review conducted in 2021 [47].

Although male-to-female ratio estimates vary from country to country, ASD prevalence is consistently higher among boys than girls worldwide. The higher prevalence of ASD diagnoses in males compared to females is a complex issue that continues to be studied. Several factors may contribute to this disparity, including biological differences between males and females that influence the development or manifestation of ASD. These differences may involve genetic, hormonal, or neurobiological factors. Also, diagnostic bias may play a role. Historically, ASD traits may have been more readily recognized and diagnosed in males than in females. Furthermore, social and behavioral differences may skew prevalence rates. Males and females may exhibit ASD-related behaviors differently. Social expectations and norms might also influence how symptoms are perceived and reported. In addition, there may be gaps in research focusing on ASD in females, potentially leading to an underestimation of prevalence among females. Understanding these factors requires ongoing research and a nuanced approach to diagnosis and intervention across sexes [23,48].

The mean age at diagnosis in our study was 4.5 ± 2.5 years. Ages from 2 to 6 years old show the highest prevalence of ASD diagnosis confirmation, with the peak at 3 years old. Similar results have been reported in the USA, where the median age of ASD diagnosis confirmation ranged from 36 to 63 months (CDC). This study, conducted in two regions of Poland from 2010 to 2014 and focusing on children aged 0 to 16 years, found that the highest prevalence of ASD was reported among children aged 4 to 7 years old [35]. In Germany, the highest prevalence of ASD has been observed among children aged 6 to 11 years [26], while in India, it has been reported among children aged 6 to 10 years [41].

Our study revealed that the ASD prevalence was significantly higher in urban areas (3/1000) as compared to rural areas (1/1000). There are several factors that may contribute to the higher prevalence of ASD in urban areas compared to rural areas: for instance, access to services [46,49]. Urban areas typically have better access to specialized healthcare services, including diagnostic facilities and therapeutic interventions for ASD. This can lead to more accurate diagnoses and higher reported prevalence rates. Second, awareness and education may play a significant role: Urban populations often have greater awareness about ASD among healthcare professionals, educators, and families [50,51,52]. This can result in earlier identification and diagnosis of ASD cases. Third, population density is another important factor: higher population density in urban areas can lead to more opportunities for interactions and assessments, increasing the likelihood of ASD diagnoses being made.

In addition, environmental factors are significant contributors: urban environments may have higher levels of environmental factors like air pollution [11,53], endocrine-disruptive agents [54], and heavy metals [55,56] that could potentially influence neurodevelopment and contribute to the prevalence of ASD. The results of the following research are consistent with the results of studies conducted in Taiwan [44] and Vietnam [38], where most children with ASD were from urban areas. Nevertheless, this was in contrast to another study conducted across rural, urban, and tribal populations in India, where ASD prevalence was significantly higher among the rural population as compared to urban and tribal populations [40]. Finally, migration may also influence the findings: Families with children diagnosed with ASD may move from rural to urban areas in search of better services and support, which can contribute to higher prevalence rates in urban settings [57]. Overall, these factors collectively contribute to the observed higher prevalence of ASD in urban areas compared to rural areas. In turn, understanding the possible biological causes of geographic variations of ASD can serve as a proxy for suggesting the proper personalized treatment strategy [58].

The main strength of this study is that it is based on existing administrative databases of records of out-patient medical establishments related to the child population aged 0–18 years. Another strength of this study is the consistent process of ASD case identification and diagnosis confirmation across the RA, where all suspected cases of ASD were assessed by the same team of child psychiatrists and developmental pediatricians.

## 5. Limitations

The assessment of ASD prevalence in this study is based on records covering approximately 80% of the child population aged 0–18 years in RA. The exclusion of the remaining 20% could introduce bias if these children and adolescents exhibit different ASD prevalence rates or characteristics compared to those included in the study. This study relied on existing administrative databases from public outpatient medical facilities under the jurisdiction of governmental authorities, thereby excluding data from private clinics. Consequently, children and adolescents who exclusively received care at private clinics were not included in this study, which may lead to an underestimation of the true ASD prevalence in the RA child population.

## 6. Conclusions

ASD prevalence in RA demonstrates marked geographic and sex disparities, with significantly higher rates in urban regions and among males. Therefore, these findings underscore the importance of ongoing ASD prevalence studies in RA to identify trends, explore potential cause-and-effect relationships, and enhance healthcare system management, among other factors. The findings from this study could aid national and regional policymakers in health, education, and other sectors to devise or enhance effective strategies and services for children and adolescents with ASD, as well as their families.

## Figures and Tables

**Table 1:** ASD prevalence estimates per 1000 children and adolescents aged 0–18 years registered in RA, 2021.

Population Size	Number of Diagnosed ASD Cases	Prevalence per 1000 Individuals	95% CI	Average Age of ASD Diagnosis ± SD	Sex		
					Male	Female	M:F
536,659	1217	2.3	2.1–2.4	4.5 ± 2.5	83.5%	16.5%	5.1:1

**Table 2:** ASD prevalence estimates per 1000 children and adolescents (by sex) aged 0–18 years registered in RA, 2021.

Sex	Population Size	Number of ASD Cases	Prevalence/1000, 95% CI	Prevalence Ratio, 95% CI
Boys	284,429	1016	3.6 (3.4–3.8)	4.2 (3.6–4.9)
Girls	252,230	201	0.8 (0.7–0.9)	

**Table 3:** ASD prevalence estimates per 1000 children and adolescents aged 0–18 years registered in 10 marzes and Yerevan city of RA, 2021.

Yerevan and All Marzes of RA	Population Size	Number of Diagnosed	Prevalence/1000 (95% CI)	Average Age of ASD Diagnosis ± SD	Sex		
					Male	Female	M:F
Yerevan	172,473	632	3.7 (3.4–3.9)	4.61 ± 2.69	83.9%	16.1%	5.2:1
Aragatsotn	16,821	30	1.8 (1.1–2.4)	4.70 ± 2.90	76.7%	23.3%	3.3:1
Ararat	47,730	86	1.8 (1.4–2.2)	4.66 ± 2.56	87.2%	12.8%	6.8:1
Armavir	49,240	102	2.1 (1.7–2.5)	4.44 ± 2.48	81.4%	18.6%	4.4:1
Vayots Dzor	7761	11	1.4 (0.6–2.3)	4.73 ± 3.20	54.5%	45.5%	1.2:1
Gegharkunik	30,916	63	2.0 (1.7–2.5)	3.41 ± 1.75	87.3%	12.7%	6.9:1
Kotayk	53,300	62	1.2 (1.1–1.7)	4.69 ± 2.29	85.5%	14.5%	5.9:1
Lori	52,226	84	1.6 (1.3–2.0)	4.77 ± 2.34	83.3%	16.7%	5.0:1
Syunik	20,408	23	1.1 (0.9–1.5)	3.87 ± 1.56	87.0%	13.0%	6.7:1
Tavush	18,998	42	2.2 (1.5–2.9)	4.74 ± 2.50	78.6%	21.4%	3.7:1
Shirak	66,786	82	1.2 (1.0–1.5)	4.41 ± 2.27	82.9%	17.1%	4.9:1

**Table 4:** Prevalence (per 1000) of autism spectrum disorder among children and adolescents aged 0–18 years by RA regions, 2021.

RA Regions	Prevalence (95% CI)	Prevalence Ratio (PR *) (95% CI)
Yerevan	3.7 (3.4–3.9)	
Aragatsotn	1.8 (1.1–2.4)	2.0 (1.4–2.9)
Ararat	1.8 (1.4–2.2)	2.0 (1.6–2.5)
Armavir	2.1 (1.7–2.5)	1.7 (1.4–2.2)
Gegharkunik	2.0 (1.7–2.5)	1.8 (1.4–2.4)
Lori	1.6 (1.3–2.0)	2.3 (1.8–2.9)
Kotayk	1.2 (1.1–1.7)	3.1 (2.4–4.0)
Syunik	1.1 (0.9–1.5)	3.3 (2.2–5.0)
Shirak	1.2 (1.0–1.5)	3.1 (2.4–3.8)
Vayots Dzor	1.4 (0.6–2.3)	2.6 (1.4–4.7)
Tavush	2.2 (1.5–2.9)	1.7 (1.2–2.3)

* Results for PRs are considered statistically significant when the CI excludes PR = 1.0.

**Table 5:** Age at ASD diagnosis in Yerevan and marzes of RA.

Yerevan and All Marzes of RA	Age at ASD Diagnosis ± SD	Minimum Age at ASD Diagnosis	Maximum Age at ASD Diagnosis
Yerevan	4.61 ± 2.69	1	16
Aragatsotn	4.70 ± 2.90	1	13
Ararat	4.66 ± 2.56	1	16
Armavir	4.44 ± 2.48	1	13
Vayots Dzor	4.73 ± 3.20	1	10
Gegharkunik	3.41 ± 1.75	1	9
Kotayk	4.69 ± 2.29	1	11
Lori	4.77 ± 2.34	1	13
Syunik	3.87 ± 1.56	2	7
Tavush	4.74 ± 2.50	1	11
Shirak	4.41 ± 2.27	1	14

**Table 6:** ASD prevalence estimates per 1000 children and adolescents aged 0–18 years registered in urban and rural areas in RA, 2021.

All 10 Marzes and Yerevan City of of RA	Population Size	Number of Diagnosed Cases	Prevalence/1000, 95% CI	Prevalence Ratio, 95%CI	Sex		
					Male	Female	M:F
Urban areas	340,242	1029	3.0 (2.8–3.3)	2.9 (2.5–3.4)	84.3%	15.7%	5.4:1
Rural areas	196,417	188	1.0 (0.86–1.14)		79.3%	20.7%	3.8:1

**Table 7:** Age at ASD diagnosis in urban and rural areas of RA, 2021.

Yerevan and All Marzes of RA	Age at ASD Diagnosis ± SD	t-Statistic, *p*-Value	Minimum Age at ASD Diagnosis	Maximum Age at ASD Diagnosis
Urban areas	4.58 ± 2.56	t = 1.551, *p* = 0.121	1	16
Rural areas	4.27 ± 2.44		1	16

## Data Availability

The data presented in this study are available on request from the corresponding author due to ethical reasons.

## References

[1] ICD 11. Autism Spectrum Disorder. https://icd.who.int/browse11/lm/en#/http://id.who.int/icd/entity/437815624.

[2] Yenkoyan K., Grigoryan A., Fereshetyan K., Yepremyan D. (2017). Advances in understanding the pathophysiology of autism spectrum disorders. Behav. Brain Res..

[3] Mkhitaryan M., Avetisyan T., Yeritsyan H., Harutyunyan H., Yenkoyan K. (2025). Unraveling the Genetic and Environmental Risk Factors of Autism Spectrum Disorder Through a Case-Control Study in Armenia. Health Sci. Rep..

[4] Lyall K., Croen L., Daniels J., Fallin M.D., Ladd-Acosta C., Lee B.K., Park B.Y., Snyder N.W., Schendel D., Volk H. (2017). The Changing Epidemiology of Autism Spectrum Disorders. Annu. Rev. Public Health.

[5] Sauer M.A.K., Stanton M.J., Hans M.S., Grabrucker A.M., Grabrucker A.M. (2021). Autism Spectrum Disorders: Etiology and Pathology. Autism Spectrum Disorders.

[6] Colvert E., Tick B., McEwen F., Stewart C., Curran S.R., Woodhouse E., Gillan N., Hallett V., Lietz S., Garnett T. (2015). Heritability of Autism Spectrum Disorder in a UK Population-Based Twin Sample. JAMA Psychiatry.

[7] Tick B., Bolton P., Happé F., Rutter M., Rijsdijk F. (2016). Heritability of autism spectrum disorders: A meta-analysis of twin studies. J. Child Psychol. Psychiatry.

[8] Bölte S., Girdler S., Marschik P.B. (2019). The contribution of environmental exposure to the etiology of autism spectrum disorder. Cell. Mol. Life Sci..

[9] Modabbernia A., Velthorst E., Reichenberg A. (2017). Environmental risk factors for autism: An evidence-based review of systematic reviews and meta-analyses. Mol. Autism.

[10] Ng M., de Montigny J.G., Ofner M., Do M.T. (2017). Environmental factors associated with autism spectrum disorder: A scoping review for the years 2003–2013. Facteurs environnementaux associés au trouble du spectre de l’autisme: étude de délimitation portant sur les années 2003 à 2013. Health Promot. Chronic Dis. Prev. Can..

[11] Roberts A.L., Lyall K., Hart J.E., Laden F., Just A.C., Bobb J.F., Koenen K.C., Ascherio A., Weisskopf M.G. (2013). Perinatal air pollutant exposures and autism spectrum disorder in the children of Nurses’ Health Study II participants. Environ. Health Perspect..

[12] Yenkoyan K., Mkhitaryan M., Bjørklund G. (2024). Environmental risk factors in autism spectrum disorder: A narrative review. Curr. Med. Chem..

[13] Idring S., Magnusson C., Lundberg M., Ek M., Rai D., Svensson A.C., Dalman C., Karlsson H., Lee B.K. (2014). Parental age and the risk of autism spectrum disorders:Findings from a Swedish population-based cohort. Int. J. Epidemiol..

[14] Manzouri L., Yousefian S., Keshtkari A., Hashemi N. (2019). Advanced Parental Age and Risk of Positive Autism Spectrum Disorders Screening. Int. J. Prev. Med..

[15] Wu S., Wu F., Ding Y., Hou J., Bi J., Zhang Z. (2017). Advanced parental age and autism risk in children: A systematic review and meta-analysis. Acta Psychiatr. Scand..

[16] Mkhitaryan M., Avetisyan T., Mkhoyan A., Avetisyan L., Yenkoyan K. (2024). A case–control study on pre-, peri-, and neonatal risk factors associated with autism spectrum disorder among Armenian children. Sci. Rep..

[17] Lampi K.M., Lehtonen L., Tran P.L., Suominen A., Lehti V., Banerjee P.N., Gissler M., Brown A.S., Sourander A. (2012). Risk of autism spectrum disorders in low birth weight and small for gestational age infants. J. Pediatr..

[18] Losh M., Esserman D., Anckarsäter H., Sullivan P.F., Lichtenstein P. (2012). Lower birth weight indicates higher risk of autistic traits in discordant twin pairs. Psychol. Med..

[19] Lordan R., Storni C., De Benedictis C.A., Grabrucker A.M. (2021). Autism Spectrum Disorders: Diagnosis and Treatment. Autism Spectrum Disorders.

[20] Chiarotti F., Venerosi A. (2020). Epidemiology of Autism Spectrum Disorders: A Review of Worldwide Prevalence Estimates Since 2014. Brain Sci..

[21] (2013). WHO Autism Spectrum Disorders & Other Developmental Disorders. https://www.who.int/publications/i/item/9789241506618.

[22] (2025). WHO Autism. https://www.who.int/news-room/fact-sheets/detail/autism-spectrum-disorders.

[23] Loomes R., Hull L., Mandy W.P.L. (2017). What Is the Male-to-Female Ratio in Autism Spectrum Disorder? A Systematic Review and Meta-Analysis. J. Am. Acad. Child Adolesc. Psychiatry.

[24] Maenner M.J., Shaw K.A., Bakian A.V., Bilder D.A., Durkin M.S., Esler A., Furnier S.M., Hallas L., Hall-Lande J., Hudson A. (2023). Prevalence and Characteristics of Autism Spectrum Disorder Among Children Aged 8 Years—Autism and Developmental Disabilities Monitoring Network, 11 Sites, United States, 2020. MMWR Surveill. Summ..

[25] Idring S., Lundberg M., Sturm H., Dalman C., Gumpert C., Rai D., Lee B.K., Magnusson C. (2015). Changes in prevalence of autism spectrum disorders in 2001–2011: Findings from the Stockholm youth cohort. J. Autism Dev. Disord..

[26] Bachmann C.J., Gerste B., Hoffmann F. (2018). Diagnoses of autism spectrum disorders in Germany: Time trends in administrative prevalence and diagnostic stability. Autism.

[27] Public Health Agency of Canada (2018). Autism Spectrum Disorder Among Children and Youth in Canada 2018. A Report of the National Autism Spectrum Disorder Surveillance System. https://www.canada.ca/content/dam/phac-aspc/documents/services/publications/diseases-conditions/autism-spectrum-disorder-children-youth-canada-2018/autism-spectrum-disorder-children-youth-canada-2018.pdf.

[28] Public Health Agency of Canada (2022). Autism Spectrum Disorder: Highlights from the 2019 Canadian Health Survey on Children and Youth. https://www.canada.ca/content/dam/phac-aspc/documents/services/publications/diseases-conditions/autism-spectrum-disorder-canadian-health-survey-children-youth-2019/autism-spectrum-disorder-canadian-health-survey-children-youth-2019.pdf.

[29] ICD 10. Pervasive Developmental Disorders. https://icd.who.int/browse10/2019/en#/F84.

[30] Ministry of Health (2007). Regarding the Approval of Forms of Adult Outpatient Medical Card, Children’s Outpatient Medical Supervision Card, Newborn Replacement Card and Pre-Birth Patronship Sheet. (N 1752-Ն). https://www.arlis.am/DocumentView.aspx?docid=45412.

[31] Roman-Urrestarazu A., van Kessel R., Allison C., Matthews F.E., Brayne C., Baron-Cohen S. (2021). Association of Race/Ethnicity and Social Disadvantage With Autism Prevalence in 7 Million School Children in England. JAMA Pediatr..

[32] Pérez-Crespo L., Prats-Uribe A., Tobias A., Duran-Tauleria E., Coronado R., Hervás A., Guxens M. (2019). Temporal and Geographical Variability of Prevalence and Incidence of Autism Spectrum Disorder Diagnoses in Children in Catalonia, Spain. Autism Res..

[33] Valenti M., Vagnetti R., Masedu F., Pino M.C., Rossi A., Scattoni M.L., Mazza M., EAGLE GROUP (2019). Register-based cumulative prevalence of autism spectrum disorders during childhood and adolescence in central Italy. Epidemiol. Biostat. Public Health.

[34] Delobel-Ayoub M., Saemundsen E., Gissler M., Ego A., Moilanen I., Ebeling H., Rafnsson V., Klapouszczak D., Thorsteinsson E., Arnaldsdóttir K.M. (2020). Prevalence of Autism Spectrum Disorder in 7–9-Year-Old Children in Denmark, Finland, France and Iceland: A Population-Based Registries Approach Within the ASDEU Project. J. Autism Dev. Disord..

[35] Skonieczna-Żydecka K., Gorzkowska I., Pierzak-Sominka J., Adler G. (2017). The Prevalence of Autism Spectrum Disorders in West Pomeranian and Pomeranian Regions of Poland. J. Appl. Res. Intellect. Disabil..

[36] Qiu S., Lu Y., Li Y., Shi J., Cui H., Gu Y., Li Y., Zhong W., Zhu X., Liu Y. (2020). Prevalence of autism spectrum disorder in Asia: A systematic review and meta-analysis. Psychiatry Res..

[37] Sun X., Allison C., Wei L., Matthews F.E., Auyeung B., Wu Y.Y., Griffiths S., Zhang J., Baron-Cohen S., Brayne C. (2019). Autism prevalence in China is comparable to Western prevalence. Mol. Autism.

[38] Hoang V.M., Le T.V., Chu T.T.Q., Le B.N., Duong M.D., Thanh N.M., Tac Pham V., Minas H., Bui T.T.H. (2019). Prevalence of autism spectrum disorders and their relation to selected socio-demographic factors among children aged 18–30 months in northern Vietnam, 2017. Int. J. Ment. Health Syst..

[39] Magen-Molho H., Harari-Kremer R., Pinto O., Kloog I., Dorman M., Levine H., Weisskopf M.G., Raz R. (2020). Spatiotemporal distribution of autism spectrum disorder prevalence among birth cohorts during 2000–2011 in Israel. Ann. Epidemiol..

[40] Raina S.K., Chander V., Bhardwaj A.K., Kumar D., Sharma S., Kashyap V., Singh M., Bhardwaj A. (2017). Prevalence of Autism Spectrum Disorder among Rural, Urban, and Tribal Children (1–10 Years of Age). J. Neurosci. Rural Pract..

[41] Poovathinal S.A., Anitha A., Thomas R., Kaniamattam M., Melempatt N., Anilkumar A., Meena M. (2016). Prevalence of autism spectrum disorders in a semiurban community in south India. Ann. Epidemiol..

[42] Mohammadi M.R., Ahmadi N., Khaleghi A., Zarafshan H., Mostafavi S.A., Kamali K., Rahgozar M., Ahmadi A., Hooshyari Z., Alavi S.S. (2019). Prevalence of Autism and its Comorbidities and the Relationship with Maternal Psychopathology: A National Population-Based Study. Arch. Iran. Med..

[43] Gabunia M., Zirakashvili M., Nadareishvili V., Kim Y.S., Leventhal B.L., Stevanovic D. (2025). Prevalence of autism spectrum disorder in Georgia (country): A two-phase population-based study among 8–10-year-olds. Adv. Autism.

[44] Lai D.C., Tseng Y.C., Hou Y.M., Guo H.R. (2012). Gender and geographic differences in the prevalence of autism spectrum disorders in children: Analysis of data from the national disability registry of Taiwan. Res. Dev. Disabil..

[45] Doenyas C., Ekici B., Unay Ö.S., Gönen İ., Tatlı B. (2021). Autism in Turkey: Demographics, behavior problems, and accompanying medical conditions in a sample of Turkish youth with autism spectrum disorder. Int. J. Dev. Disabil..

[46] Metwally A.M., El-Din E.M.S., Sami S.M., Abdelraouf E.R., Sallam S.F., Elsaeid A., El-Saied M.M., Ashaat E.A., Fathy A.M., El-Hariri H.M. (2025). Mapping autism in Egypt: Population-based insights into prevalence, risk determinants, and severity among children aged 1–12 years. Mol. Autism.

[47] Zeidan J., Fombonne E., Scorah J., Ibrahim A., Durkin M.S., Saxena S., Yusuf A., Shih A., Elsabbagh M. (2022). Global prevalence of autism: A systematic review update. Autism Res..

[48] Giarelli E., Wiggins L.D., Rice C.E., Levy S.E., Kirby R.S., Pinto-Martin J., Mandell D. (2010). Sex differences in the evaluation and diagnosis of autism spectrum disorders among children. Disabil. Health J..

[49] Antezana L., Scarpa A., Valdespino A., Albright J., Richey J.A. (2017). Rural Trends in Diagnosis and Services for Autism Spectrum Disorder. Front. Psychol..

[50] Hsu Y.H., Chen C.W., Lin Y.J., Li C.Y. (2023). Urban-Rural Disparity in the Incidence of Diagnosed Autism Spectrum Disorder in Taiwan: A 10-Year National Birth Cohort Follow-up Study. J. Autism Dev. Disord..

[51] Mello M.P., Goldman S.E., Urbano R., Hodapp R.M. (2016). Services for Children with Autism Spectrum Disorder: Comparing Rural and Non-Rural Communities. Educ. Train. Autism Dev. Disabil..

[52] Murphy M.A., Ruble L.A. (2012). A Comparative Study of Rurality and Urbanicity on Access to and Satisfaction with Services for Children with Autism Spectrum Disorders. Rural. Spec. Educ. Q..

[53] Rahman M.M., Shu Y.H., Chow T., Lurmann F.W., Yu X., Martinez M.P., Carter S.A., Eckel S.P., Chen J.C., Chen Z. (2022). Prenatal Exposure to Air Pollution and Autism Spectrum Disorder: Sensitive Windows of Exposure and Sex Differences. Environ. Health Perspect..

[54] Bjørklund G., Mkhitaryan M., Sahakyan E., Fereshetyan K., A Meguid N., Hemimi M., Nashaat N.H., Yenkoyan K. (2024). Linking Environmental Chemicals to Neuroinflammation and Autism Spectrum Disorder: Mechanisms and Implications for Prevention. Mol. Neurobiol..

[55] Harutyunyan A.A., Harutyunyan H.A., Yenkoyan K.B. (2021). Novel Probable Glance at Inflammatory Scenario Development in Autistic Pathology. Front. Psychiatry.

[56] Lee K.S., Min W.K., Choi Y.J., Jin S., Park K.H., Kim S. (2023). The Effect of Maternal Exposure to Air Pollutants and Heavy Metals during Pregnancy on the Risk of Neurological Disorders Using the National Health Insurance Claims Data of South Korea. Medicina.

[57] Lauritsen M.B., Astrup A., Pedersen C.B., Obel C., Schendel D.E., Schieve L., Yeargin-Allsopp M., Parner E.T. (2014). Urbanicity and autism spectrum disorders. J. Autism Dev. Disord..

[58] Yenkoyan K., Ounanian Z., Mirumyan M., Hayrapetyan L., Zakaryan N., Sahakyan R., Bjørklund G. (2024). Advances in the Treatment of Autism Spectrum Disorder: Current and Promising Strategies. Curr. Med. Chem..

